# Magnetically controlled growing rods in the management of early onset scoliosis: a systematic review

**DOI:** 10.1186/s13018-022-03200-7

**Published:** 2022-06-11

**Authors:** Filippo Migliorini, Wai On Chiu, Raffaele Scrofani, Wai Kwong Chiu, Alice Baroncini, Giorgio Iaconetta, Nicola Maffulli

**Affiliations:** 1 Department of Orthopaedics, Trauma, and Reconstructive Surgery, University Clinic Aachen, RWTH Aachen University Hospital, Pauwelsstraße 31, 52074 Aachen, Germany; 2 Master Program of Biomedical Engineering, Faculty of Medicine, RWTH Aachen University, Aachen, Germany; 3 Department of Neurosurgery, University Hospital of Salerno, Fisciano, Italy; 4 MBBS School of Medicine, Jinan University, Guangzhou, China; 5 Department of Medicine, Surgery and Dentistry, University of Salerno, 84081 Baronissi, Italy; 6 School of Pharmacy and Bioengineering, Keele University Faculty of Medicine, ST4 7QB Stoke-on-Trent, England, UK; 7 Centre for Sports and Exercise Medicine, Barts and the London School of Medicine and Dentistry, Queen Mary University of London, E1 4DG London, England, UK

**Keywords:** Spine, Deformity, Early onset scoliosis, Magnetically controlled growing rods, MCGR

## Abstract

**Background:**

Early onset scoliosis (EOS) presents in patients younger than 10 years. Magnetically controlled growing rods (MCGR) were developed as an outpatient distraction system for EOS, allowing to avoid multiple surgeries. This systematic review investigated the efficacy and feasibility of MCGR in EOS.

**Methods:**

This systematic review was conducted according to the PRISMA guidelines. PubMed, Google scholar, Embase, and Scopus were accessed in May 2022. All the clinical trials which investigate the role of MCGR for early onset scoliosis were accessed. Only studies reporting data in patients younger than 10 years with a preoperative Cobb Angle greater than 40° were eligible. The following data was extracted at baseline and at last follow-up: mean kyphosis angle, overall mean Cobb angle, mean *T*1–*S*1 length. Data from complication were also collected.

**Results:**

Data from 23 clinical studies (504 patients) were included in the present study. 56% (282 of 504) were females. The average length of the follow-up was 28.9 ± 16.0 months. The mean age of the patients was 8.7 ± 1.9 years old. The mean BMI was 17.7 ± 7.6 kg/m^2^. The mean kyphosis angle had reduced by the last follow-up (*P* = 0.04), as did the overall mean Cobb angle (*P* < 0.0001), while the overall *T*1–*S*1 length increased (*P* = 0.0002). Implant-associated complications, followed by spinal alignment failure, wound healing ailments, pulmonary complications, progressive trunk stiffness, persistent back pain, and fracture.

**Conclusion:**

The management of EOS remains challenging. The current evidence indicates that MCGR may be effective to distract the spine and model the curve in EOS.

## Introduction

Early onset scoliosis (EOS) presents in patients younger than 10 years [1, 2]. EOS is classified according to the age of the patient at the start of the deformity as early (0–5 years old) or late (> 5 years old) onset scoliosis [3–5]. Surgery in patients with EOS aims to correct the deformity avoiding complications improving the patient’s long-term health-related quality of life [6–9]. Concomitant neuromuscular, congenital, or syndromic scoliosis are associated with greater morbidity and mortality [3, 10, 11]. If left untreated, EOS may lead to cardiopulmonary and neurological complications [12, 13]. Given their growth-friendly nature, traditional growing rods (TGR) have been used to treat EOS. However, surgical distraction every 6–8 months is required, increasing the risk of complication [6, 14–16]. Magnetically controlled growing rods (MCGR) were developed as an outpatient distraction system, allowing to avoid multiple surgeries [17, 18]. Spinal distraction using MCGR is performed by placing a manual magnetic external remote controller over the internal magnet. Next, the rotation of the magnet within the rod is induced by external magnetic field leads to extension of rod. At the moment, the MAGEC (Magnetic Expansion Control; NuVasive; San Diego; USA) is the only magnetically regulated growing rod system [6, 18]. In Europe, the system was licensed in 2009 and it was approved by the FDA in 2017 [6]. Although this methodology reduces the number of surgical interventions, it has been associated with several complications [15, 19].

This systematic review investigated the efficacy and feasibility of MCGR in EOS. The focus of the present study was on kyphosis, Cobb angle, *T*1–*S*1 length, and complications associated with MCGR.

## Material and methods

### Search strategy

This systematic review was conducted according to the Preferred Reporting Items for Systematic Reviews and Meta-Analyses: the PRISMA guidelines [20]. The PICO algorithm was preliminary pointed out:

- *P (Population)* Early onset scoliosis;
- *I (Intervention)* MCGR;
- *C (Comparison)* efficacy and feasibility;
- *O (Outcomes)* reliability and safety.

### Data source

Two authors (**;**) independently accessed PubMed, Google scholar, Embase, and Scopus in April 2022. The following keywords were used in combination: *early onset scoliosis, scoliosis, spine, young, children, childhood, magnetic controlled growing rods, MCGR, three dimensional spinal deformity correction, apical control of vertebrae.* The same authors independently screened the resulting titles and abstract. The full-text of the articles which matched the topic was accessed. A cross reference of the bibliographies was also performed by hand. Disagreement was debated and solved by a third author (**).

### Eligibility criteria

All the clinical trials which investigate the role of MCGR for early onset scoliosis were accessed. Given the authors language capabilities, articles in English, German, Italian, French, Spanish, and Chinese were eligible. Only level I–III of evidence articles, according to Oxford Centre of Evidence-Based Medicine [21], were considered. Only studies reporting data in patients younger than 10 years with a preoperative Cobb Angle greater than 40° were eligible. Only studies with minimum 9 months’ follow-up are considered. Both single and double rod MCGR were considered. Reviews, letters, expert opinion, editorials, and comments were not eligible. Animal, cadaveric, and biomechanics studies were excluded. Only articles reporting quantitative data under the outcomes of interest were considered for inclusion. Missing data under the outcomes of interest warranted the exclusion from this study.

### Data extraction

Two authors (**;**) independently performed data extraction. Studies generalities (author, year, design, length of the follow-up) were extracted, as were patient demographic (size, gender, mean age, and BMI). Data of the patient baseline characteristics were extracted. The following data was extracted at baseline and at last follow-up: mean kyphosis angle, overall mean Cobb angle, mean *T*1–*S*1 length. Data from complication were also collected.

### Methodology quality assessment

For the methodology quality assessment, the Coleman Methodology Score (CMS) was used [22]. Each of the included studies was evaluated under several items, such as the population size, length of follow-up, number of surgical approaches, diagnosis, surgical techniques with description, outcomes and related assessing procedure, and patient recruitment. The CMS evaluated every included article in a value from 0 to 100. A mean overall value > 60 points is considered as ‘satisfactory’.

### Data synthesis

The statistical analyses were performed using the IBM SPSS Software version 25. For descriptive statistics, mean and standard deviation were used. For continuous variables, the mean difference (MD) effect measure was adopted. The *t*-test was used to assess whether the change of variables from baseline to last follow-up were statistically significant, with values of *P* < 0.05 considered satisfactory. The rate of adverse events was evaluated as frequency (%).

## RESULTS

### Search result

**Fig. 1:**
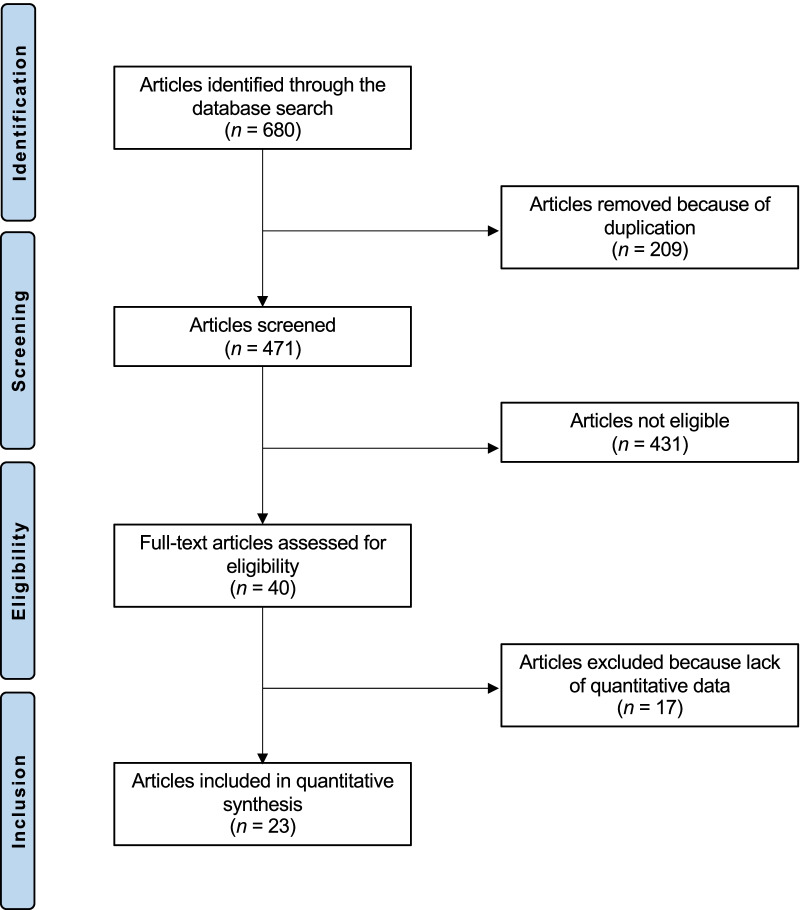
Flow chart of the literature search

The literature search resulted in 663 articles, 209 of them were excluded from this study as they were duplicated. Another 431 articles were excluded since they did not fulfil the preferred eligibility criteria: language limitation (*n* = 68), type of studies (*n* = 167), type of analysis (*n* = 178) and revision settings (*n* = 18). Finally, 41 articles were rejected as they did not provide qualitative data under outcomes of interest. This left 23 investigations for the present study. The literature search results are shown in Fig. 1.

### Methodological quality assessment

The limited study size, along with the retrospective design of most of the included studies represent important limitations of this study. The average length of the follow-ups was acceptable in most studies. Diagnosis and surgical approach were well-described in most articles. The rehabilitation process was barely reported. Outcome measures and timing of assessment were satisfactory defined. The procedures for assessing outcomes and subject selection were often biased and not adequately described. The CMS for the articles was 66/100, attesting the good quality of the methodology assessment (Table 1).

**Table 1:** CMS

Endpoint	Mean value
*Part A: Only one score to be given for each of the 7 sections*	
1. Study size: number of patients	2.78
2. Mean follow-up	4.70
3. Surgical approach	8.26
4. Type of study	6.52
5. Description of diagnosis	2.20
6. Descriptions of surgical technique	7.17
7. Description of postoperative rehabilitation	2.61
*Part B: Scores may be given for each option in each of the 3 sections if applicable*	
1. Outcome criteria	
Outcome measures clearly defined	1.91
Timing of outcome assessment clearly stated	2.00
Use of outcome criteria that has reported reliability	3.00
General health measure included	2.50
2. Procedure of assessing outcomes	
Participants recruited	5.00
Investigator independent of surgeon	3.75
Written assessment	3.00
Completion of assessment by patients themselves with minimal investigator assistance	1.00
3. Description of subject selection process	
Selection criteria reported and unbiased	4.91
Recruitment rate reported > 80%	4.80
Recruitment rate reported < 80%	0.00

### Patient demographics

504 patients were included in the present study. 56% (282 of 504) were females. The average length of the follow-up was 28.9 ± 16.0 months. The mean age of the patients was 8.7 ± 1.9 years old. The mean BMI was 17.7 ± 7.6 kg/m^2^. Studies generalities and patient baseline is shown in Table 2.

**Table 2:** Generalities and patient baseline of the included studies (please define the abbreviations)

Author, year	Journal	Design	Follow-up (months)	Patients (n)	Mean age	Eligibility criteria
Akbarnia et al. [23]	Spine Deformity	Prospective	10	14	8.8	1. EOS of any aetiology; 2. clear indication for an operative intervention; 3. Minimum 3 outpatient distractions were done
Burstein et al. [24]	J Spine	Prospective	31	22	8.8	1. EOS at any aetiology; 2. Cobb’s angle > 40 degree, and/or progression > 5 deg., and/or failed non-operative treatment; 3. FU minimum 2 years
Cheung et al. [25]	Lancet	Prospective	19	5	11.0	1. Remain growth potential; 2. Treated with MCGR for 24 months
Cheung et al. [26]	Neurosurgery	Retrospective	73.2	31	10.1	1. Minimum 4-year FU for post-implantation of single and dual MCGR
Cheung et. al. [27]	Spine Deformity	Prospective	24	10	8.2	1. No prior treatment to spinal deformity;2. At least 2-year FU after primary MCGR insertion
Dahl et al. [28]	J Orthop Surg	Retrospective	22.8	19	9.7	1. Treated with MCGRs at a single situation; 2. primary surgery
Dannawi et al. [29]	Bone Joint J	Prospective	15	34	8.0	1. EOS from any cause; 2. Failed non-operative treatment with bracing or observative; 3. Progression of the curvature of > 10 deg. over 6-month period with Cobb angle > 40°; 5. any evidence of infection
Di Silvestre et al. [30]	Eur Spine J	Retrospective	34.8	17	14.5	1. Adolescent idiopathic scoliosis; 2. Thoracic curve with minimum preoperative Cobb’s angle 90 deg.; 3. at least 2.5 years FU; 4. Aged between 11 and 17 years old; 5. No preoperative treatment with halo-traction/serial corrective Risser’s plasters
Doany et al. [31]	Spine	Retrospective	34.3	44	6.7	1. EOS of any aetiology; 2. age less than 10 years at index surgery; 3. Major curve greater than or equal to 30 deg.; 4. No history of spine surgery before growing rod insertion; 5. At least 12-month postoperative follow-up
Heydar et al. [32]	Spine	Prospective	9	18	7.3	1. EOS of any aetiology; 2. Cobb's angle equals or greater than 40 deg.; 3. Rising Cobb’s angle by 10 deg. 4. *T*1–*T*12 < 22 cm; 5. Younger 10 years; 6. Accepted implantation for min. period of 9 months; 7. Consent from parents; 8. MAGEC implantation as primary surgery
Hickey et al. [33]	Eur Spine J	Prospective	23	8	4.5	1. Inserted magnetic controlled growth rods (MAGEC) for management of EOS with minimum of 23 months follow-up
Keskinen et al. [34]	Eur Spine J	Retrospective	12	50	7.0	1. Diagnosis of EOS; 2. Surgery before age of 11 years; 3. Minimum 30° major curve by Cobb angle; 4. Thoracic spinal height < 22 cm; 5. At least 1-year postoperative follow-up
Kwan et al. [19]	Spine	Retrospective	37	30	7.2	1. Progressive spinal deformity
La Rosa et al. [35]	J Pediatr Orthop	Prospective	27	10	7.2	1. EOS was diagnosed before age of 5; 2. Patients was initially managed with cast and braces until value of curvature > 40 deg
Lebon et al. [36]	Eur Spine J	Retrospective	18.4	30	9.1	1. Failed conservative treatment/revision of GR; 2 follow-ups longer than 12 months
Pepke et al. [37]	Der Orthopäede	Retrospective	24	21	9.2	1. Patients were under 10 years old; 2. scoliotic curve > 40°; 3. primary spine surgery; 4. any congenital, syndromic/neuromuscular scoliosis
Ridderbusch et al. [38]	J Paed Orthop	Retrospective	21.1	24	8.9	1. EOS at any aetiology; 2. Major curve > 40 Deg. 3. at least 12moths FU; 4. at least 3 lengthening steps
Skov et al. [7]	Int Orthop	Prospective	67.2	38	10.2	1. Progressive EOS of all aetiology are treated with Cody Bünger concept; 2. Minimum 2 years FU; 3. any salvage/complex spine procedure with/without Cody Bünger concept; 4. longer than 2 years EOS treatment
Studer et al. [39]	J Children orthop	Prospective	25	30	9.4	1. First 30 patients underwent MCGR treatment
Teoh et al. [40]	Spine J	Retrospective	48	8	8.2	1. Minimum 44 months of FU
Thompson et al. [41]	Bone Joint J	Prospective	22.4	19	9.1	1. Diagnosed with progressive thoracic/thoracolumbar scoliosis
Wijdicks et al. [14]	Spine Deformity	Retrospective	36	18	9.9	1. Skeletal immaturity; 2. Progressive scoliosis; 3. Major curve > 45 deg
Yoon et al. [42]	Spine	Prospective	30	6	7.5	1. EOS secondary to neuromuscular disease

### Imaging

The mean kyphosis angle had reduced by the last follow-up (− 10.9°; *P* = 0.04), as did the overall mean Cobb angle (− 31.6; *P* < 0.0001), while the overall *T*1–*S*1 length increased (+ 27.1 mm; *P* = 0.0002). The mean kyphosis, Cobb angle, and *T*1–*S*1 length are shown in greater detail in Table 3.

**Table 3:** Improvement from baseline

Endpoints	Baseline	Last FU	MD	*P*
Mean kyphosis angle (°)	42.7 ± 9.5	31.8 ± 7.4	− 10.9	0.04
Overall mean Cobb angle (°)	68.2 ± 10.8	36.6 ± 8.5	− 31.6	< 0.0001
Cobb angle of patients who received MCGR as primary procedure (°)	66.0 ± 7.2	39.7 ± 4.0	− 26.3	0.01
Cobb angle of patients who received MCGR as reivision procedure (°)	45.7 ± 1.2	40.0 ± 3.5	− 5.7	0.08
Cobb angle of patients who received dual rod MCGR (°)	63.0 ± 9.9	36.0 ± 5.7	− 27.0	0.04
Cobb angle of patients who received single rod MCGR (°)	67.5 ± 0.7	41.0 ± 4.2	− 26.5	0.04
Overall *T*1–*S*1 length (mm)	183.6 ± 13.5	210.7 ± 10.8	27.1	0.0002
*T*1–*S*1 length of patients who received MCGR as primary procedure (mm)	240.0 ± 35.4	290.5 ± 24.7	50.5	0.05
*T*1–*S*1 length of patients who received MCGR as revision procedure (mm)	289.5 ± 23.3	307.0 ± 29.7	17.5	0.08
*T*1–*S*1 length of patients who received single rod MCGR (mm)	301.0 ± 8.5	334.5 ± 17.7	33.5	0.06
*T*1–*S*1 length of patients who received dual rod MCGR (mm)	299.3 ± 3.8	322.0 ± 38.2	22.7	0.3

*FU* follow-up

### Complications

Implant-associated complications, including proximal screw pull out from the rod, pull out of the rod, re-operation for trimming of the prominent rod, connector breakage, completed breakage, detachment of pedicle screw hand/screw misplaced, and screw/plug loosening were the most common ones. The following spinal alignment were found: coronal imbalance, proximal junctional kyphosis, pelvic obliquity, secondary lumbar scoliosis. Delayed wound healing, wound dehiscence, and infections were seldom reported. Pulmonary complications, such as pneumonia, pulmonary embolism, pleural effusion, and progressive trunk stiffness, persistent back pain and fracture occurred rarely (Table 4).

**Table 4:** Overall complications

Complications	Frequency	Reference
Proximal screw out of the rod	29% (4 of 14)	[33]
Loss initial height	21% (3 of 14)	[23]
Proximal pull out of the hooks	19% (11 of 59)	[41]
Screw/plug loosening	18% (10 of 57)	[7, 28]
Reoperation for rod malposition	17% (1 of 6)	[42]
Fail distraction	12% (25 of 216)	[14, 19, 26, 28, 33, 34, 38, 39]
Painful out patient distraction	12% (7 of 60)	[36]
Connector breakage	11% (4 of 38)	[7]
Progressive trunk shiftiness	11% (2 of 18)	[14]
complete blockage/rod breakage	10% (20 of 197)	[7, 14, 19, 27–29, 33, 34, 39, 40]
Proximal junctional kyphosis	9% (16 of 183)	[7, 24, 26, 28, 33, 36, 39]
Infection	8% (11 of 142)	[7, 19, 23–27, 29, 34, 39, 40]
Coronal imbalance	8% (3 of 38)	[7]
Pleural effusion	6% (1 of 17)	[30]
Back pain	5% (3 of 56)	[14]
Detachment of pedicle screw hand/screw misplaced	5% (2 of 38)	[7]
Pelvic obliquity	5% (1 of 19)	[28]
Pulmonary complications	4% (3 of 68)	[7]
Fracture	4% (2 of 56)	[14]
Delayed wound healing	3% (2 of 61)	[39]
Secondary scoliosis	3% (1 of 30)	[36]
Wound dehiscence	3% (1 of 30)	[36]
Pulmonary embolism	3% (1 of 30)	[36]

The number of events is reported on the number of observations reported by each study

## Discussion

The management of EOS remains challenging. MCGR is effective to distract the spine and model the curve in EOS, and is associated with a low rate of complication in the short term. Compared to the increase lengthening method of TGR, MCGR is a noninvasive procedure that avoids periodic invasive distraction procedures, exposing the patients to less anaesthesia episodes, rate of postoperative complications, reducing the physical and psychological burden of the young patients. Moreover, MCGR can be performed as outpatients, which may lead to a marked reduction in costs compared to the traditional TGR. However, is necessary to ascertain mid to long research whether the good preliminary outcomes persist [19, 43, 44].

The most common sagittal plane abnormality remains thoracic lordosis or thoracic hypokyphosis. To measure the magnitude of frontal plane deformity in scoliosis the Cobb angle is commonly used. The Cobb angle is used to determine in a relatively easy fashion the degree of curvature of the spine [45–47]. The Cobb angle is determined in posteroanterior radiographies. To assess the Cobb angle, the beginning and end of the spinal curve must be identified. Two lines, one tangential to the cranial endplate of the beginning vertebrae and one tangential to the caudal endplate of the last vertebrae are drawn. In a person with a straight spine, these lines would be parallel. In people with spinal curvatures, perpendicular lines are drawn from these lines until they intersect. The lines follow the inclination of the vertebrae and are angled. The Cobb angle is the angle at the point of intersection [48, 49]. The overall mean Cobb angle and the dorsal kyphosis at last follow-up reduced considerably of 31.6° and 10.9°, respectively. The *T*1–*S*1 vertebral lengthening at last follow-up also improved significantly of 27 mm. These data suggest that MCGR is effective and comparable with TGR [50, 51]**.** One limitation of the Cobb angle is that people can stand four to six degrees off when taking the measurement [47, 52]. This can mean the difference between bracing and surgery, making such measurements critical [53, 54]. Additionally, this measurement identifies the spine as a two-dimensional object on radiographies, when in fact the spine exists in three dimensions [55, 56]. The Cobb angle does not consider the twisting of the spine that often accompanies the development of a side-to-side curvature [57, 58]. A patient might have a small Cobb angle, but a severely twisted spine [59, 60].

Overall, a total of 124 complications were reported in 504 patients (25%). The foremost frequent complications were: proximal screw out of the rod, loss initial height, proximal pull out of the hooks, screw/plug loosening, and reoperation for trimming of outstanding rod. Failure of distraction, connexion breakage, complete blockage/rod breakage, proximal junctional humpback. Thought the rate of complications was high, this value is lower than what observed following TGR, at approximately 46–55% [43, 44, 61].

MCGR is initially more expensive compared to the TGR; however, the lower number of surgeries required, the outpatient regime, and the lower rate of complications results in a lower burden in the mid to long term in favour of MCGR [62, 63]. The cumulative costs of MCGR are approximately 50% greater than TGR at 1 year follow-up; however, they are lower of about 17% at 5 years follow-up [64].

This study has several limitations. The retrospective nature of the present study represents an important limitation, which increase the risk of selection bias. The study size was limited and the length of the follow-up was too short in most included studies. Surgical approach, eligibility criteria, and rehabilitation protocols were often biased and biased. Outcome measures and timing of assessment were often defined, providing moderate reliability. General health measures were seldom described. The timing of the evaluation of the results was often biased. Future high-quality studies involving a larger number of patients and longer follow-up are required to proper validate MCGR in the clinical setting. Given these limitations, data from the present study must be considered carefully.

## Conclusion

The management of EOS remains challenging. The current evidence indicates that MCGR may be effective to distract the spine and model the curve in EOS.

## Data Availability

The data underlying this article are available in the article and in its online supplementary material.

## Abbreviations

EOS: Early onset scoliosis
MCGR: Magnetically controlled growing rods
MAGEC: Magnetic expansion control
BMI: Body mass index
CMS: Coleman methodology score
MD: Mean difference
TGR: Traditional growing rods
